# New System for the Classification of Epiphyseal Separation of the Coracoid Process: Evaluation of Nine Cases and Review of the Literature

**DOI:** 10.1155/2020/9749515

**Published:** 2020-10-23

**Authors:** Takamitsu Mondori, Yoshiyuki Nakagawa, Shimpei Kurata, Shuhei Fujii, Takuya Egawa, Kazuya Inoue, Yasuhito Tanaka

**Affiliations:** ^1^Department of Orthopaedics, Uda City Hospital, Nara Shoulder & Elbow Center, 815 Hagiwara, Haibara, Uda, Nara 633-0298, Japan; ^2^Department of Orthopaedics, Nara Medical University, 840 Shijyo, Kashihara, Nara 634-8521, Japan; ^3^Department of Orthopaedics, Nara Prefecture Seiwa Medical Center, 1-14-16 Mimuro, Sangou-cho, Ikoma-gun, Nara 636-0802, Japan; ^4^Department of Orthopaedics, Okanami General Hospital, 1734 Ueno Kuwamachi, Iga, Mie 518-0842, Japan

## Abstract

*Objectives and Design*. Epiphyseal separation of the coracoid process (CP) rarely occurs in adolescents. In this retrospective case series, we reviewed the data of nine patients treated at our center and those of 28 patients reported in the literature. This injury can be classified into three types according to the injured area: Type I, base including the area above the glenoid; Type II, center including the coracoclavicular ligament (CCL); and Type III, tip with the short head of the biceps and coracobrachialis, as well as the pectoralis minor. *Patients/Participants*. A total of 37 patients were included in the analysis. Data on sex, age, cause and mechanism of injury, separation type, concomitant injury around the shoulder girdle, treatment, and functional outcomes were obtained. *Main Outcome Measurements and Results*. Type I is the most common type. The cause of injury and associated injury around the shoulder girdle were significantly different between Type I, II, and III fractures. The associated acromioclavicular (AC) dislocation and treatment were significantly different between Type I and III fractures. Our new classification system reflects the clinical features, imaging findings, and surgical management of epiphyseal separation of the CP. Type I and II fractures are mostly associated with AC dislocation and have an associated injury around the shoulder girdle. Type III fractures are typically caused by forceful resisted flexion of the arm and elbow. Although the latter are best managed surgically, whether conservative or surgical management is optimal for Type I and II fractures remains controversial. *Conclusions*. We noted some differences in the clinical characteristics depending on the location of injury; therefore, we aimed to examine these differences to develop a new system for classifying epiphyseal separation of the CP. This would increase the clinicians' awareness regarding this injury and lead to the development of an appropriate treatment.

## 1. Introduction

Fractures of the coracoid process (CP) do not commonly occur, accounting for only 2%–13% of all scapular fractures and approximately 1% of all fractures [1–4]. The epiphyseal separation of the CP in the adolescent is even more uncommon [5, 6], with few cases reported in the literature [1, 4, 7–26]. This injury can complicate acromioclavicular (AC) dislocations and fractures of the coracoid in adults. Although a diagnosis of AC dislocation is easily made, the epiphyseal separation of the CP may be overlooked due to the complexity of the anatomical structures and superimposition on standard shoulder radiographs. Misdiagnosis of isolated AC dislocation, which is mainly due to damage of the coracoclavicular ligament (CCL), has a profound influence on the choice of treatment and prognosis. In order to establish an accurate diagnosis of epiphyseal separation of the CP, clinicians must have a good understanding of the pathophysiology of this injury and the location of the epiphyseal line of the CP. Therefore, the appropriate classification of this injury is necessary. Although several classification systems for coracoid fractures in adults have been proposed from previous studies, there is no available system for classifying epiphyseal separation of the CP in adolescents.

## 2. Objectives

The aim of this study was to examine the clinical characteristics associated with epiphyseal separation of the CP and propose a new classification system for this condition.

## 3. Materials and Methods

### 3.1. Participant Recruitment

The epiphyses of the coracoid close as the child reaches the age of 17–25 [11, 27]. Therefore, we recruited all published cases of patients aged below 17 years or over whose computed tomography (CT) images clearly revealed epiphyseal lesions as cases of adolescent epiphyseal separation of the CP. We retrospectively reviewed nine patients who were treated at our center and the data of 28 published cases, and we found that epiphyseal separation of the CP differs depending on the location of the injury. We hypothesized that each site may have its own characteristics. Nine patients with epiphyseal separation of the CP were treated immediately after obtaining an injury at our center between 1989 and 2019 (Table 1). All patients underwent follow-up examinations for >1 year and were directly examined at our center at the final observation. All patients were included in this retrospective study, regardless of treatment type or concomitant injuries. Additionally, we identified another 28 cases by review of the literature that provided sufficient case details (Table 2). All procedures performed in studies involving human participants were in accordance with the ethical standards of the 1964 Helsinki Declaration and its later amendments or comparable ethical standards. Informed consent was obtained from all study participants. This study was approved by the Ethics Committee of Uda City Hospital (approval number: R2-002).

### 3.2. Data Extraction and Analysis

The medical records from our center and previously published studies were retrospectively reviewed to extract the data regarding sex, age, cause and mechanism of injury, separation type, concomitant injury around the shoulder girdle, treatment, and functional outcomes.

**Figure 1:**
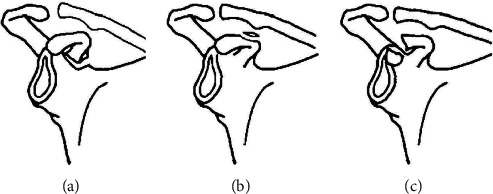
The proposed classification of epiphyseal separation of the coracoid process along with the anteroposterior radiograph and/or three-dimensional computed tomography reconstruction. Type I: the base including the area above the glenoid; Type II: the center with the coracoclavicular ligament; and Type III: the tip including the short head of the biceps and coracobrachialis, in addition to the pectoralis minor.

**Figure 2:**
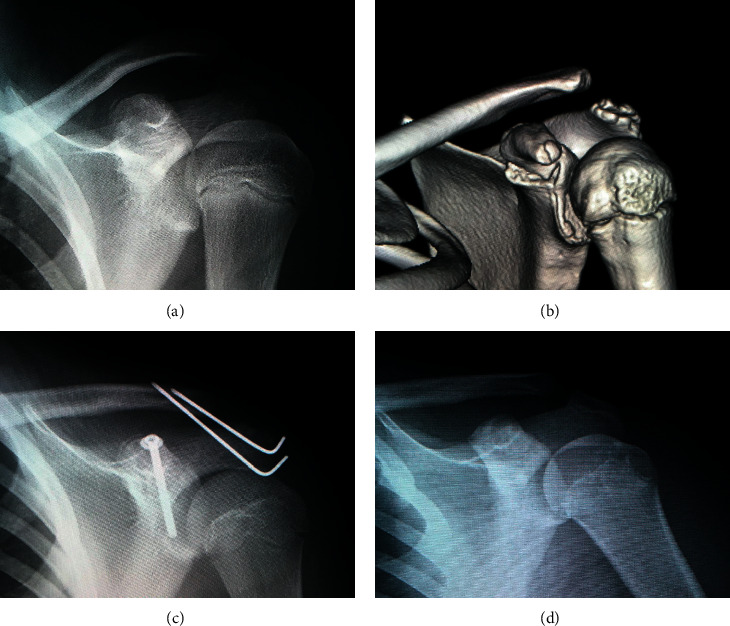
Case 6: representative anteroposterior radiograph: (a) three-dimensional computed tomography reconstruction and (b) Type I injury with an associated acromioclavicular dislocation. Anteroposterior radiograph immediately after the surgery (c) and anteroposterior radiograph three years after the surgery (d).

**Figure 3:**
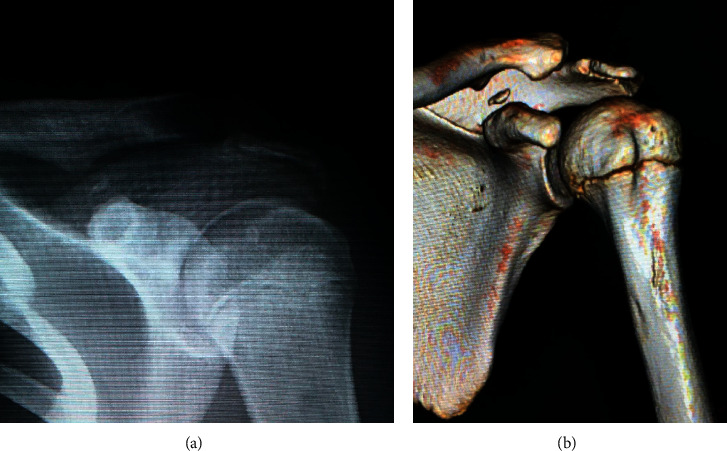
Representative anteroposterior radiograph of case 9: three-dimensional computed tomography reconstruction (a) and type II injury with an associated acromioclavicular dislocation (b).

**Figure 4:**
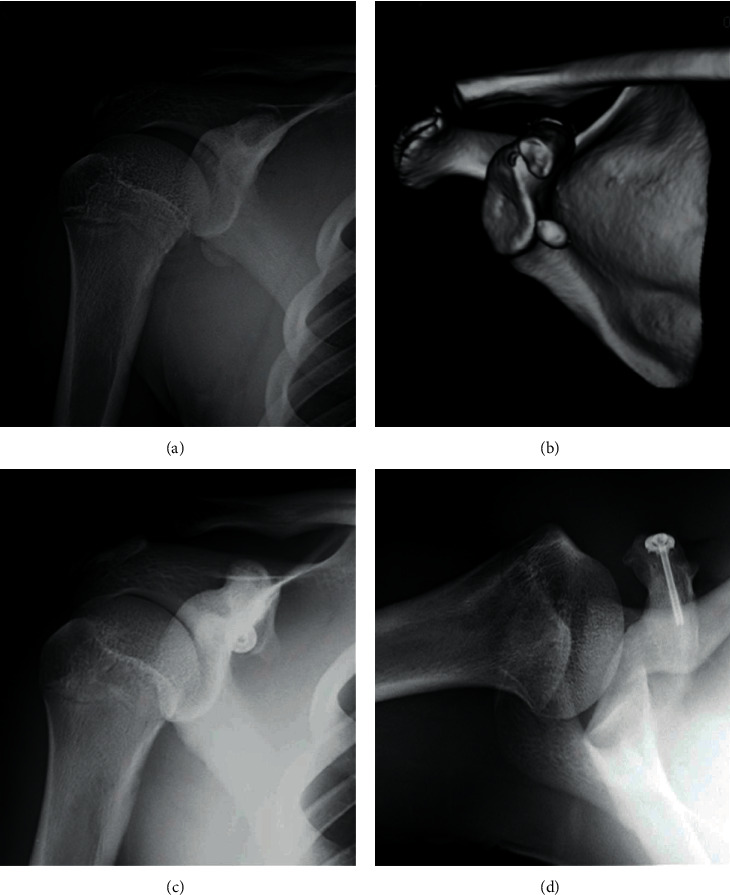
Representative anteroposterior radiograph: three-dimensional computed tomography reconstruction (a), Type III injury (b), anteroposterior radiograph two years postoperatively (c), and axillary radiograph two years postoperatively (d). This image was used with permission from Kurume University Medical Center and Dr. K. Nakama [21].

In these 37 patients, separation occurred at the base of the CP. The separation occurred above the glenoid in 28 (76%) patients, at the center with CCL in 6 (16%), and at the tip of the short head of the biceps and coracobrachialis or the pectoralis minor in 3 (8%). The differences identified in the epiphyseal separation of the CP lesions were classified depending on the location of the injury (Figure 1): Type I, the base including the area above the glenoid (Figure 2); Type II, the center with CCL (Figure 3); and Type III, the tip including the short head of the biceps and coracobrachialis in addition to the pectoralis minor (Figure 4).

The three types of fractures were compared statistically in terms of sex, age, cause and mechanism of injury, concomitant injury around the shoulder girdle, concomitant AC dislocation, and treatment (surgery or conservative therapy) and functional outcome (excellent, good/fair, or poor).

In this study, the method used for evaluating functional outcome was not standardized. Therefore, with regard to the clinical results at the time of final observation described in the article, the absence of (1) pain, (2) limited range of motion, and (3) inability to return to sports were considered as excellent. If one of the abovementioned items were reported by the patient, the results were considered to be good/fair. If two or more of the abovementioned items were reported by the patient, the results were considered to be poor.

### 3.3. Statistical Analysis

Statistical analysis was performed using the StatMate IV software for Windows (version IV; ATMS ISBN:978-4-90-430722-9, 2009, Japan). The Kruskal–Wallis test with Bonferroni/Dunn correction was used to compare sex, age, cause and mechanism of injury, concomitant injury around the shoulder girdle, concomitant AC dislocation, treatment, and functional outcomes; the chi-square test or Fisher's exact test was used to compare sex, cause and mechanism of injury, concomitant injury around the shoulder girdle, concomitant AC dislocation, treatment, and functional outcomes.

## 4. Results

The average age at the time of injury was 14.4 years (Type I, 14.0 years; Type II, 15.7 years; Type III, 16.7 years). Among the total population, except for one example that was not described, 33 (91.7%) were men and three (8.3%) were women. In cases of Type I injury, 21 (75%) patients had associated injuries around the shoulder girdle with the following breakdown: 19 (90.4%), AC dislocation; 1 (4.8%), clavicle distal end fracture; and 1 (4.8%), a combination of lateral clavicular epiphyseal separation and rupture of the CCL. In Type II, five patients had associated injuries around the shoulder girdle, of whom four (80%) had AC dislocation and one (20%) had double fracture of the clavicle. All Type III cases were isolated injuries.

In the Type I group, the mechanism of injury in three patients was unknown. Among the patients whose mechanism of injury was identified, 14 (56%) experienced falling on the shoulder, 8 (32%) had a direct trauma to the shoulder, and 3 (12%) had forceful resisted flexion of the arm and elbow. In Type II, the mechanism of injury was unknown in one patient; among the patients with known mechanism of injury, falling on the shoulder was reported in four (80%) and direct trauma to the shoulder in one (20%). All cases of Type III injury were caused by forceful resisted flexion of the arm and elbow. Conservative treatment was carried out in 21 (75%) patients with Type I injury, and surgical therapy was administered in 7 (25%) patients. All surgical cases of Type I injury associated with AC dislocation were repaired by screw fixation of the coracoid and AC joint using a Kirschner wire (K-wire). The functional outcomes were good or excellent for both methods. For Type II injuries, conservative treatment was used in four (66.7%) patients, and surgery was performed in two (33.3%). In the Type II group, one of the four (25%) patients treated conservatively had a poor functional outcome and two (100%) patients who underwent surgery showed an excellent functional outcome. Surgical therapy was used in all patients with Type III injury, with excellent clinical results. The cause of injury and associated injury around the shoulder girdler were significantly different between Type I, II, and III cases. The associated AC injury and treatment were significantly different between Type I and Type III cases (Table 3).

## 5. Discussion

**Figure 5:**
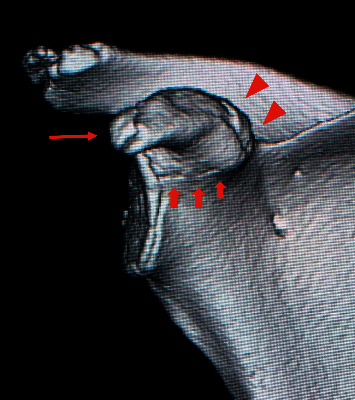
Representative three-dimensional computed tomography image of a normal epiphyseal line around the coracoid of an 11-year-old girl. A normal coronoid process has three epiphyseal lines: the base (broad arrows), center (triangular arrows), and tip (narrow arrow).

Due to the rarity of epiphyseal separation of the CP, clinicians' understanding and knowledge regarding clinical management of this condition is limited [5, 6]. Although epiphyseal separations of the CP are similar to CP fractures in terms of the clinical presentation and mechanism of injury, the imaging features that lead to the diagnosis, the healing form, and the prognosis differ depending on the age and the presence or absence of the epiphysis. The coracoid has four (or three) main centers where ossification can occur: the base and body of the process, the center of the process at the point of attachment of the CCL, and the tip [11, 20, 21, 23, 28]. The epiphyseal nucleus of the body of the coracoid appears 1 year after birth, and that of the base of the coracoid appears at the age of 7–10 years; soon it is in unison with the emerged scapula body; therefore, there are three epiphyseal plates between each epiphyseal nucleus [28] (Figure 5). The center of the process is the site of insertion of the CCL [29], while the tip is the site of insertion of the conjoint tendon (the short head of the biceps and coracobrachialis, as well as the pectoralis minor). During development, the coracoid and epiphyseal plate at the base and tip fuse by the age of 17 years, while the epiphyseal plate at the center fuses by the age of 25 years [11, 27]. Prior to epiphyseal closure, the ligament and muscle attachments are often stronger than the epiphyseal plate. This means that injury to the epiphyseal plate is more common in younger individuals [4, 11, 23, 30]. In this study, three sites were damaged during the epiphyseal separations of the CP, and their positions also corresponded to the three epiphyseal plates.

A number of several classification systems for coracoid fractures in adults have been reported. In 1995, Eyres et al. classified these fractures into five types based on the location of fracture (Type I, tip or epiphyseal fracture; Type II, mid-process; Type III, basal fracture; Type IV, superior body of scapula involved; and Type V, extension into glenoid fossa) [1]. Later, Ogawa et al. proposed a new classification system dividing the CP into two distinct locations based on the CCL attachment: Type I fractures are located behind the ligaments, while Type II fractures are located in front of the ligaments [2]. To date, there has been no classification system proposed for epiphyseal separation of the CP in adolescents. Fractures classified as Type I according to our system are equivalent to Type I fractures of Ogawa et al. and Type III, IV, and V fractures of the classification of Eyres et al. Fractures classified as Type II by our system are equivalent to Type II fractures of the system of Ogawa et al. and Type I and II fractures of Eyres et al. However, our Type II classification is not equivalent to any type of previous systems. Although the mechanism of injury for Type II fractures combined with AC dislocation is the same as that of isolated AC dislocation, epiphyseal separation can occur rather than disruption of the CCL in adolescents because the epiphyseal plate is weaker than the CCL.

Epiphyseal separation of the CP is usually diagnosed by obtaining plain shoulder radiographs consisting of three views. Special radiograms are required in order to make a definitive diagnosis: 30° cephalad roentgenogram [7], 45° to 60° cephalad tilt [31], or abduction view that clearly scans the CP without overlapping other bone structures [9, 16]. However, CT, especially three-dimensional CT, and magnetic resonance imaging are usually necessary because of the limitations of plain radiography [1, 14, 18]. Comparison of CT data from the healthy side may help in the accurate diagnosis of this condition. Duerr et al. [19] reported an exceedingly rare case of combined lateral clavicular epiphyseal separation (or AC joint dislocation), the base of coracoid separation, and rupture of the coracoclavicular ligaments, a so-called “triple injury.” In cases of injury I that involves double disruption of the superior shoulder suspensory complex in the case of >100% displaced distal clavicle separation, careful scrutiny of radiographs is important to ensure correct identification of the CCL, AC ligament, and other sites. Thus, given the challenges in the diagnosis of epiphyseal separation of the CP from imaging investigations, our proposal of classifying these fractures into three types will facilitate the correct diagnosis of this condition from such examinations. By recognizing in advance that epiphyseal separation of the CP can occur in three places, attention can be drawn to the epiphyseal line of the CP when making a diagnosis.

There have been three mechanisms of injury reported for coracoid fractures in adults [14]: direct trauma to the anterolateral aspect of the shoulder [32], direct trauma to the shoulder girdle usually caused by a fall or blow with the arm in the adducted position that leads to AC dislocation [33], and forceful resisted flexion of the arm and elbow leading to a strong pull of the muscles inserting into the coracoid, pectoralis minor, and coracobrachialis [10, 14, 26, 34]. In the present study, most Type I and II injury cases were caused by direct trauma to the anterolateral aspect of the shoulder with involvement of the shoulder girdle; AC dislocation without associated CCL tear accounted for two-thirds of the cases. Interestingly, a clear difference in the mechanism of injury of Types I and II was observed from that of Type III injury. Because Type III injuries occur from overuse or forceful resisted flexion of the arm and elbow [20–22], the factor of fatigue fracture is considered to be involved.

In our center, only Type I and Type II cases were reported. In cases with AC joint dislocation, surgical treatment is recommended because the presence of AC joint dislocation will cause a dysfunction in the future, and conservative treatment is recommended for patients who refused to undergo surgery. Hence, surgery was performed in 3 of 7 patients with AC joint dislocation. The type of surgical procedure performed was the same as that conducted in previous studies, with screw fixation of the coracoid process and percutaneous fixation of the AC joint using a Kirschner wire. All operative cases had excellent outcomes. All Type I and II patients who have undergone surgery underwent surgical treatment to cure AC joint dislocation in previous studies. However, the review of the literature revealed no clear advantage of surgery over conservative treatment because most patients with Type I and II were treated conservatively with good/excellent outcomes [11, 26]. More studies are required to clarify the advantages or differences in outcomes between surgical treatment and conservative treatment. By contrast, all patients with Type III injuries reported in the literature were treated surgically by reattaching the fragment or conjoined tendon and had good/excellent outcomes. Surgical therapy involving rigid fixation can result in early improvements in the range of motion and return to training and normal physical or sports activities [20–22].

This study has some limitations. The sample size was small, particularly for Type II and III fractures. We were unable to clearly determine the difference between Type I and Type II cases for each item. If the number of Type II cases increases, a difference may be found. The study is retrospective in nature; hence, it was difficult to confirm all images of cases in the literature. In the future, data on the characteristics of this injury according to type must be obtained by conducting a prospective study.

## 6. Conclusions

We propose a classification system for epiphyseal separation of the CP based on the location of ossification. Our new system includes consideration of clinical features, imaging findings, and surgical management. The application of the system revealed that Type I injuries occur predominantly in younger patients compared with Type II and III. Type I and II injuries are most commonly associated with AC joint dislocation and associated injury of the shoulder girdle. Type III injuries are most commonly caused by forceful resisted flexion of the arm and elbow, and surgical therapy offers the best outcomes. However, the management for Type I and II injuries remains controversial because both approaches appear to be effective. Further investigations are required to ascertain the optimal method.

## Figures and Tables

**Table 1:** Characteristics of patients with epiphyseal separation of the coracoid process treated in our center.

	Location of separation	Age	Sex	Cause of injury	Mechanism of injury	Associated injury^*∗*^	Treatment	Functional outcome
1	Base (I)	15	M	Fall	Fall on the shoulder	AC dislocation (II)	ACJ: K-wiring coracoid: screw fixation	Excellent

2	Base (I)	15	M	Fall from bicycle	Fall on the shoulder	AC dislocation (II)	Conservative (sling 4 weeks)	Excellent

3	Base (I)	14	M	Rugby	Direct trauma by tackle	AC dislocation (III)	Conservative (sling 4 weeks)	Excellent

4	Base (I)	11	M	Fall	Fall on the shoulder	Clavicle distal end fixation	Coracoid: screw fixation	Excellent

5	Base (I)	14	M	Soccer	Fall on the shoulder	AC dislocation (III)	ACJ: K-wiring coracoid: screw fixation	Excellent

6	Base (I)	11	F	Judo	Fall on the shoulder	AC dislocation (III)	ACJ: K-wiring coracoid: screw fixation	Excellent

7	Base (I)	16	M	Fall	Fall on the shoulder	AC dislocation (III)	Conservative (sling 4 weeks)	Excellent

8	Center (II)	16	M	Motorcycle	Fall on the shoulder	Clavicle double fixation	Clavicle: K-wire and soft wire fixation	Good after infection

9	Center (II)	17	M	Motorcycle	Fall on the shoulder	AC dislocation (II)	Conservative (sling 4 weeks)	Excellent

AC, acromioclavicular; ACJ, acromioclavicular joint; F, female; M, male. ^*∗*^The numbers in parentheses indicate the grade of AC dislocation (II: subluxation of AC joint; III: complete dislocation of AC joint).

**Table 2:** Characteristics of patients with epiphyseal separation of the coracoid process identified from the literature review.

	Location of separation	Publish year	Author	Age	Sex	Cause of injury	Mechanism of injury	Associated injury^*∗*^	Treatment	Functional outcome
1	Tip (III)	1971	Benton J	19	M	Tennis	Overuse or forceful resisted flexion of the arm	—	Conjoined tendon reattach	Excellent

2	Base (I)	1975	Protass JJ	17	M	Football	Unknown	AC dislocation (III)	Conservative	Unknown

3	Base (I)	1975	Protass JJ	14	M	Fall off the bicycle	Unknown	AC dislocation (II)	Conservative	Unknown

4	Center (II)	1977	Montgomery SP	15	M	Football	Fall on the shoulder	AC dislocation (III)	Epiphysis reattached by a nonabsorbable suture	Excellent

5	Center (II)	1977	Montgomery SP	15	M	Bike accident	Unknown	AC dislocation (III)	Conservative (sling 4 weeks)	Poor

6	Base (I)	1982	Bernard TN	13	M	Football	Direct trauma	AC dislocation (III)	Conservative (AC immobilizer 4 weeks)	Excellent

7	Base (I)	1982	Bernard TN	15	M	Football	Fall on the shoulder	AC dislocation (III)	Conservative (AC immobilizer 6 weeks)	Excellent

8	Base (I)	1982	Bernard TN	17	M	Motorcycle	Direct trauma	AC dislocation (III)	ACJ: K-wiring, coracoid: screw fixation	Good

9	Base (I)	1986	Taga I	9	F	Unknown	Unknown	—	Conservative (Velpeau bandage 4 weeks)	Excellent

10	Base (I)	1990	Martin-Herrero T	16	M	Free skating	Forceful resisted flexion of the arm	AC dislocation (III)	Conservative (Desault bandage 4 weeks)	Excellent

11	Base (I)	1990	Martin-Herrero T	17	M	Judo	Fall on the shoulder	AC dislocation (?)	Conservative (Watson–Jones bandage 3 weeks)	Excellent

12	Base (I)	1995	Combalia A	12	M	Soccer	Fall on the shoulder	AC dislocation (III)	Conservative (Robert–Jones bandage 4 weeks)	Excellent

13	Base (I)	1995	Eyres KS	17	M	Folk-lift overturn	Trapping the arm	AC dislocation (III)	Conservative (broad arm sling)	Unknown

14	Base (I)	1996	Cottalorda J	15	M	Judo	Fall on the shoulder	—	Conservative	Excellent

15	Base (I)	1998	Holst AK	13	M	Fall	Fall on the shoulder	—	Conservative (broad arm sling 2 weeks)	Excellent

16	Base (I)	1999	Naraen A	11	M	Archery	Overuse or forceful resisted flexion of the arm	—	Conservative (sling 2 weeks)	Excellent

17	Base (I)	2009	Dipaora M	15	M	American football	Direct trauma by tackle	AC dislocation (II)	Conservative (sling)	Excellent

18	Center (II)	2009	Leijnen M	16	M	Fall off motorcycle	Fall on the shoulder	—	Conservative	Excellent

19	Base (I)	2010	Jettoo P	12	M	Fall from high place	Fall on the shoulder	AC dislocation (III)	ACJ: K-wiring coracoid: screw fixation	Excellent

20	Tip (III)	2011	Nakama K	16	M	Gymnastic (frying ring)	Overuse or forceful resisted flexion of the arm	—	Coracoid: screw fixation	Excellent

21	Base (I)	2012	Alsey KJ	14	?	Rugby	Direct trauma by tackle	—	Conservative (sling 4 weeks)	Excellent

22	Base (I)	2012	Chitre AR	13	M	Ski	Fall on the shoulder	—	Conservative	Excellent

23	Base (I)	2012	Chitre AR	15	M	Wheelbarrow race	Fall on the shoulder	—	Conservative	Excellent

24	Base (I)	2014	Pedersen V	14	M	Ice-hockey	Direct trauma by tackle	AC dislocation (II)	Conservative (sling)	Excellent

25	Center (II)	2016	Ito T	15	F	Judo	Direct trauma	AC dislocation (III)	ACJ: K-wiring coracoid: soft anchor fixation	Excellent

26	Tip (III)	2016	Archik S	15	M	Cricket	Overuse or forceful resisted flexion of the arm	—	Coracoid: screw fixation	Excellent

27	Base (I)	2018	Cross GWV	15	M	Rugby tackled violently	Direct trauma by tackle	AC dislocation (III)	Conservative (sling)	Excellent

28	Base (I)	2019	Duerr RA	12	M	Scooter accident	Direct trauma to the superolateral shoulder	Epiphyseal separation of the distal clavicle, CCL tear (triple injury)	Coracoid: screw fixation CCL and ACL repair	Excellent

AC, acromioclavicular; ACJ, acromioclavicular joint; CCL, coracoclavicular ligament; F, female; M, male. ^*∗*^The numbers in parentheses indicate the grade of AC dislocation (II: subluxation of AC joint; III: complete dislocation of AC joint; ?: unidentified).

**Table 3:** Results of statistical analysis.

	Kruskal–Wallis test, Bonferroni/Dunn correction			Chi-square test/Fisher's exact test		
	I-II	I–III	II-III	I-II	I–III	II-III
Age	NS	NS	NS	—	—	—
Sex (male/female)	NS	NS	NS	NS	NS	NS
Cause of injury	NS	*p* < 0.05	*p* < 0.05	—	—	—
Associated injury around the shoulder girdle (yes/no)	NS	*p* < 0.05	*p* < 0.05	NS	*p* < 0.05	*p* < 0.05
Associated injury AC dislocation (yes/no)	NS	NS	NS	NS	*p* < 0.05	NS
Treatment (surgery/conservative)	NS	*p* < 0.05	NS	NS	*p* < 0.05	NS
Functional outcome (excellent, good/fair, or poor)	NS	NS	NS	NS	0	NS

AC, acromioclavicular; NS, not significant.

## Data Availability

The data used to support the findings of this study are included within the article.
